# Effects of combined high-intensity aerobic interval training program and Mediterranean diet recommendations after myocardial infarction (INTERFARCT Project): study protocol for a randomized controlled trial

**DOI:** 10.1186/s13063-018-2529-3

**Published:** 2018-03-02

**Authors:** Sara Maldonado-Martín, Jon Ander Jayo-Montoya, Tatiana Matajira-Chia, Beatriz Villar-Zabala, Juan José Goiriena, G. Rodrigo Aispuru

**Affiliations:** 1 Department of Physical Education and Sport, Faculty of Education and Sport, Physical Activity and Sport Sciences Section, University of the Basque Country (UPV/EHU), Portal de Lasarte, 71, 01007 Vitoria-Gasteiz, Araba/Álava, Basque Country Spain; 2 Cardiology Department, Santiago Apóstol Hospital, Miranda de Ebro, Burgos, Spain; 3 Primary Care Administration of Burgos, Miranda de Ebro, Burgos, Spain; 4 Department of Physiology, Faculty of Medicine, University of the Basque Country (UPV/EHU), Leioa, Bizkaia, Basque Country Spain

**Keywords:** Exercise design, Cardiorespiratory fitness, Low-volume training, Health status

## Abstract

**Background:**

Exercise therapy has long been used for rehabilitation purposes after myocardial infarction (MI) and the benefit of regular physical exercise is also well-established. High-intensity interval training (HIIT) has been proposed to be more effective than continuous exercise for improving exercise capacity and health-related adaptations to low-volume (LV) and HIIT are also known. Furthermore, the Mediterranean diet (Mediet) has been widely reported to be a model of healthy eating for its contribution to a favorable health status and a better quality of life, reducing overall mortality. This study will investigate the effects of different HIIT programs (high-volume [HV] vs LV) and Mediet recommendations in clinical condition, cardiorespiratory fitness, biomarkers, ventricular function, and perception of quality of life after MI, and compared to an attention control group that is recommended to Mediet and physical activity without supervision sessions.

**Methods/Design:**

In this randomized controlled trial, cardiorespiratory fitness, anthropometry, central and peripheral cardiovascular variables, biochemical and nutritional condition, and quality of life will be assessed before and after 16 weeks of intervention in 177 participants diagnosed with MI type 1. All participants will be randomly (1:1:1) assigned to the attention control group or two exercise groups (Mediet recommendations plus supervised aerobic exercise two days/week: (1) HV (40 min) HIIT group and (2) LV (20 min) HIIT group.

**Discussion:**

This study will be the first clinical trial comparing the effects of two different volumes of HIIT programs with Mediet recommendations for people after MI. The results of this study will provide good evidence for physical rehabilitation in this population.

**Trial registration:**

ClinicalTrials.gov, NCT02876952. Registered on 24 August 2016.

**Electronic supplementary material:**

The online version of this article (10.1186/s13063-018-2529-3) contains supplementary material, which is available to authorized users.

## Background

The term “myocardial infarction” (MI) may have major psychological and legal implications for the individual and society. It remains the principal cause of death and disability worldwide and is a measured outcome in clinical trials [1]. Hence, coronary heart disease is a chronic condition and patients are at high risk for new events and premature death. Several evidence-based interventions can improve prognosis. Thus, lifestyle changes should be explained and proposed to the patients before discharge, including cessation of smoking, blood pressure (BP) control, advice regarding diet and weight control, and the encouragement of physical activity. Exercise therapy has long been used for rehabilitation purposes and the benefit of regular physical exercise is also well-established [2]. It is known that exercise can improve both cardiovascular and non-cardiovascular parameters, such as improved anxiety, patient self-confidence, endothelial function, exercise capacity, cardiovascular efficiency, reduction in atherogenic and thrombotic risk factors, and perception of wellbeing have been described [2–6]; it is also included as an essential component for secondary prevention [7]. Furthermore, the risks of a well-designed supervised exercise program are very low [2, 8]. The intensity of aerobic exercise training is a key issue in cardiac rehabilitation programs [9]. Intensity ranges for aerobic training prescription and design are included in several guidelines and publications regarding secondary prevention and cardiac rehabilitation [9, 10]. Aerobic fitness is recognized as a robust indicator of cardiovascular health and a well-established predictor of total and cardiovascular mortality in people with and without coronary heart disease. Direct measurements of peak oxygen uptake (VO_2peak_) and ventilatory thresholds (i.e. VT1 and VT2) are considered the gold standard references for the evaluation of aerobic metabolism function and, consequently, for aerobic exercise intensity assessment and design [9, 11]. The increase of VO_2peak_ and VTs after a period of exercise training depends on the components of the FITT principle (i.e. frequency, intensity, time or volume, and type or modality), which constitute the key to achieve a safe exercise training effect. Endurance aerobic training is typically performed as continuous training at moderate-to-high exercise intensity in steady-state conditions of aerobic energetic yield. However, high-intensity interval training (HIIT) (i.e. repeated bouts of short duration, high- to severe- or severe- to extreme-intensity exercise, separated by brief periods of lower intensity) has been proposed to be more effective than continuous exercise for improving exercise capacity [9, 12–16]. Adding to that, health-related adaptations to low-volume and high-intensity interval training (LV-HIIT) have been presented. This type of training is characterized by sessions that involve a relatively small total amount of exercise at high intensity (i.e. ≤ 10 min) [17]. Previous studies have shown that LV-HIIT is an effective and time-efficient training strategy for improving insulin sensitivity, glucose control, and biomarkers of vascular function [18] and that training-induced adaptation for high-volume (HV) moderate-intensity continuous exercise and LV-HIIT protocols are strikingly similar [19]. Although contradictory results of the effect of HIIT in cardiovascular patients have been previously presented [20], to our knowledge there are no studies that compare HIIT with different volume exercise in a population that has suffered MI and compare that to an attention control group (AC) who is receiving only physical activity recommendations.

On the other hand, the relevance of overall high-quality food patterns, rather than focus on single nutrients and foods, has emerged as a powerful paradigm to address the diet and to assess their potential cardiovascular disease preventative effects [21]. The Mediterranean diet (Mediet), representing the dietary pattern usually consumed among the populations bordering the Mediterranean Sea, has been widely reported to be a model of healthy eating for its contribution to a favorable health status and a better quality of life, reducing overall mortality from cardiovascular diseases [22].

Considering all the above-mentioned points, the INTERFARCT study is designed to investigate what effect different 16-week supervised aerobic INTERval exercise programs (HV vs LV) with Mediet recommendations will have in people after suffering an acute myocardial inFARCTion compared to AC that is recommended a Mediet and physical activity without supervision sessions.

We hypothesize that: (1) supervised HIIT will get superior improvements in cardiovascular health than only physical activity recommendations; and (2) a high-intensity and LV exercise program will get similar results compared to a high-intensity and HV exercise program. Therefore, the primary objective of this randomized controlled trial (RCT) will be to assess the effects of different programs of HIIT and Mediet recommendations on overall health, cardiorespiratory fitness, biomarkers, ventricular function, and perception of quality of life after MI compared to only physical activity recommendations. The secondary objectives will be: (1) to analyze the differences in the studied variables between the two HIIT programs (HV vs LV) with Mediet recommendations to observe the effect of exercise volume; and (2) to analyze whether a treatment with only recommendations (exercise and diet) is effective in the secondary prevention of cardiovascular disease compared to supervised exercise.

## Methods/design

### Study design

INTERFARCT is a prospective, open-label, blinded-endpoint, three-arm, parallel RCT (ClinicalTrials.gov ID: NCT02876952) and fulfills the requirements of the Standard Protocol Items: Recommendations for Interventional Trials (SPIRIT) checklist [23] (Additional file 1). The Ethics Committee of the University of the Basque Country (UPV/EHU, CEISH, 2016) and the Ethics Committee of Clinical Investigation of Burgos University Hospital (CEIC 1462) have approved the study design, protocol, and informed consent procedure. All participants have to provide written informed consent before the clinical and physiological examination. Each participant will be given the opportunity to ask questions about the involvement in the trial and to have those questions answered by a researcher associated with the trial before providing consent. After baseline measurements, the participants will be followed for 16 weeks. All follow-up examinations will be performed in the same laboratory setting and by the same researchers as in the baseline measurements. After baseline testing, they will be enrolled in the trial given a trial-specific identification (i.e. INTERFARCT-1) number (ID). Allocation consignment was performed by one of the exercise physiologists who was not involved in the recruitment process. The participants were randomized to one of the three intervention groups (Attention Control [AC], HV High-Intensity Interval Training [HV-HIIT], or LV-HIIT) stratified by sex, body mass index (BMI), and age using a block and stratified randomization method and with a randomization ratio of 1:1:1. Medical staff will be blinded to participant randomization assignment. The trial will be conducted in the Cardiology Department at Santiago Apóstol Hospital, Miranda de Ebro, Burgos, Spain. A trial-specific ID number will be used when gathering and recording information about a participant. A list containing the contact details of all participants and their corresponding ID numbers will be kept separate from all other data. All paper copy forms will be stored in a locked cabinet in the hospital facility during the trial and only researchers involved in the trial will have access. Study data will be accessible by the researchers involved in the trial and only the principal investigator and investigators in the project will have access to data analysis.

### Participants and selection criteria

The population will include 177 participants (men and women) with the diagnosis of MI according to criteria of “Third Universal definition of myocardial infarction” and clinical classification of MI type 1, called “Spontaneous myocardial infarction” [1], recruited from the cardiology services by the physician specialists. In turn, before testing protocol begins, an ergometry is performed according to the clinical history for participants’ screening to ensure there is no ongoing myocardial ischemia. After completion of the informed consent process, clinical history, blood sample for baseline analysis, electrocardiogram, and echocardiogram are obtained from all participants to evaluate the eligibility criteria. Only participants who have been managed with revascularization are included. The inclusion and exclusion criteria for the INTERFARCT study are shown in Table 1. After assessment for eligibility and consent, different clinical research members collect the baseline data necessary to complete the pre-randomization information.

**Table 1:** Inclusion and exclusion criteria for the INTERFARCT study

Inclusion criteria
- MI type I: Spontaneous MI [1], with and without ST elevation
- Effective revascularization treatment (coronary artery bypass grafting or percutaneous coronary intervention)
- Age ≥ 18 years, clinically stable on sinus rhythm
- Between six months and two years after the MI
- Left ventricular ejection fraction > 50%
- Time availability (45 min, two days a week for 16 weeks) to carry out the exercise program
Exclusion criteria
- Unstable coronary artery disease, uncontrolled hypertension, malignant ventricular arrhythmia, atrial fibrillation, exercise-induced ischemia, and ventricular failure during exercise
- Other significant medical conditions including, but not limited to: chronic or recurrent respiratory, gastrointestinal, neuromuscular, neurological, or psychiatric conditions; musculoskeletal problems interfering with exercise; severe kidney disease (creatinine clearance < 30 mL/min, calculated in accordance with Modification of Diet in Renal Disease equation [MDRD]), autoimmune or collagen vascular diseases; immunodeficiency diseases or a positive human immunodeficiency virus test; anemia (hemoglobin < 12 g/dL), bleeding disorders, chronic thrombotic disorders, or hypercoagulable states; malignancies in the past five years, with the exception of skin cancer therapeutically controlled; endocrine and metabolic disorders, including type 1 diabetes; moderate to severe peripheral artery disease (> IIa in Fontaine’s classification); any other medical condition or disease that is life-threatening or that can interfere with or be aggravated by exercise
- Any other co-morbidity with life expectancy < 1 year
- Could not perform a valid baseline exercise test - Obesity > 35 kg/m^2^
- Pregnancy or breastfeeding
- Plans to be out of the city > 2 weeks

### Measurements

**Fig. 1:**
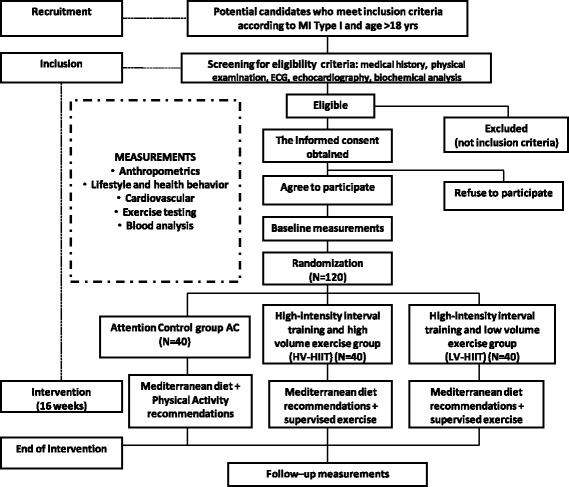
Planned *flow diagram* of the INTERFARCT study from recruitment to the end of the intervention

**Fig. 2:**
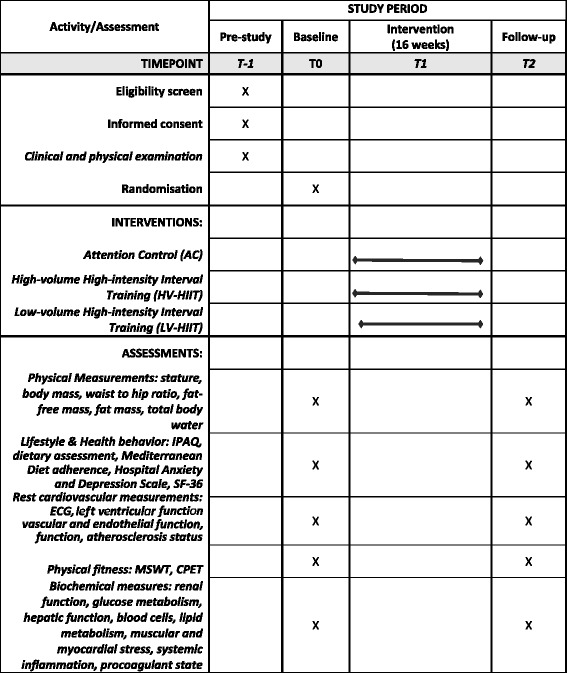
SPIRIT *figure* showing an overview of the assessment schedule at baseline and follow-up in the INTERFARCT study. IPAQ International Physical Activity Questionnaire, ECG electrocardiogram, MSWT Modified Shuttle Walking Test, CPET cardiopulmonary exercise test

The measurements used in the protocol will be taken before and after the intervention period (16 weeks). The study protocol flow of participants is outlined in Fig. 1. The post-intervention test will be scheduled the following week after finishing the 16-week intervention period. The primary outcome variable is cardiorespiratory fitness, measured through VO_2peak_. The secondary outcome variables include left ventricular function, body composition, biochemical profile, vascular endothelial function, dietary adherence, the state of depression and anxiety, and health-related quality of life. The SPIRIT figure showing the respective time points for assessments and intervention is provided in Fig. 2.

#### Anthropometry and body composition

Anthropometry includes stature (SECA 213), total body mass (SECA 869), BMI calculated as (total body mass [kg]/stature [m^2^]), and waist and hip circumferences (SECA 200) to calculate waist:hip ratio. All measurements will be taken in accordance with guidelines from the International Society for the Advancement of Kinanthropometry [24]. Furthermore, fat-free mass, total body water, and fat mass will be estimated with bioelectrical impedance analysis (Tanita, BF 350 and Tanita, BC-418 MA).

#### Lifestyle and health behavior

Participants’ health-related quality of life will be determined using the 36-item Short Form Medical Outcome Questionnaire (SF-36) [25]. It is a 36-item questionnaire that measures eight multi-item dimensions of health: physical functioning; social functioning; role limitations due to physical problems; role limitations due to emotional problems; mental health; energy/vitality; body pain; and general health perception. Scores will be coded, summed, and converted into a scale from 0 (worst possible health state) to 100 (best possible health state) for each dimension item.

Anxiety and depression levels will be assessed through the Spanish version of the Hospital Anxiety and Depression Scale (HADS), which is a 14-item self-report screening scale. The HADS consists of a seven-item anxiety subscale and a seven-item depression subscale. Each item scores on a 4-point Likert scale [26].

#### Cardiovascular measurements at rest

A 12-lead electrocardiogram, left ventricular function by echocardiography, flow-mediated dilation (FMD), and carotid intimae media thickness (CIMT) by ultrasound scanning will be the methods used to assess central and peripheral cardiovascular variables.

Echocardiography will be performed by two experienced cardiologists blinded to the participant’s group assignment. The average of these two measurements will be used in the statistical analysis [27]. Participants will be examined at rest using an ultrasound system (GE Healthcare, Vivid 9) according to standard procedures for evaluated left ventricle dimensions and diastolic and systolic functions [27]. Left ventricle end-diastolic diameter, diastolic and systolic volumes, and ventricular mass will be measured and indexed to body surface area. Assessment of left ventricle diastolic function will include peak early (E) and late (A) diastolic mitral inflow velocities, early deceleration time (EDT), early diastolic tissue Doppler velocity (e’), and E/e’ ratio. On the other hand, determination of left ventricle systolic function will be carried out by calculation of biplane ejection fraction (Simpson’s rule), cardiac output (CO), and determination of peak annulus velocity in systole (S′).

For each participant, microvascular and conduit artery function in the forearm will be assessed with non-invasive, high-resolution ultrasonography of the brachial artery, using a handheld ultrasound probe. Flow-mediated dilation, a biomarker for endothelial function, will be evaluated with high-resolution ultrasonography and performed following the International Brachial Artery Reactivity Task Force guidelines using a 2.5- to 13-MHz linear array transducer (GE Healthcare, Vivid 7) with a stereotactic probe-holding device. Conduit artery function will be assessed of FMD (endothelium-dependent response) and nitroglycerin-mediated dilation (endothelium-independent response) [28–30].

Carotid intima-media thickness (GE Healthcare, Vivid 7) will be carried out by two sonographers blinded to the intervention’s groups assignment. For each individual, mean CIMT will be defined as the average of all six segments intima-media thickness measurements in each carotid artery [31, 32]. The image analyses will be performed with validated software (eTRACK) [33] and all ultrasonographic measures will be analyzed by another blind specialist.

#### Physical fitness

Physical fitness will include the Modified Shuttle Walking Test (MSWT) and a peak, symptom-limited cardiopulmonary exercise test (CPET). The two tests will be conducted on different days. The purpose of doing a CPET and MSWT will be to evaluate the exercise responses in both physical activity modalities (i.e. walking/running and biking).

The MSWT requires the participant to walk up and down a 10-m course and it will be performed as previously described by Singh et al. [34]. Participants will walk along a flat, indoor 10-m course marked by two cones placed 0.5 m in from each end of the course. A shuttle will refer to one 10-m lap. Standardized prerecorded instructions for the test will be played from a digital recording immediately before beginning the test. The test will be externally paced, with signal beeps at regular intervals to indicate when the participant should be turning around the cone to commence the next shuttle. A triple beep will signal the next level and an increase in walking speed. Participants will commence the test at a walking speed of 0.5 m/s (level 1), allowing the participant 20 s to complete each of the three shuttles in level 1. There will be a speed increment of 0.17 m/s each minute for a maximum of 15 min. The test will be stopped when the participant can no longer maintain the required pace or is > 0.5 m from the cone before the signal beep after one opportunity to catch up or if the test is completed. Additional criteria for early termination of the test will include patient distress, dizziness, angina, or onset of severe musculoskeletal pain, failure of the heart rate (HR) to increase with exercise or attainment of 85% of the maximum HR (estimation by subtracting the person’s age from 220). The number of shuttles completed will be recorded at the completion of each test and converted to the distance walked. Before commencing the test, with the participant in a seated position, baseline HR and BP will be recorded. HR and Borg scale (scale of 6–20) will be monitored throughout the test; BP and HR continue to be recorded a further 5 min after completion of the test [35, 36].

The CPET will be performed in the upright position on an electronically braked Lode Excalibur Sport Cycle Ergometer (Groningen, The Netherlands). Testing protocol will start at 0 W with gradual increments of 10 W every minute to exhaustion with continuous ECG monitoring. The test will not be preceded by any type of warm-up and the participant will cycle at least 70 rpm. The expired gas analysis will be conducted using a commercially available system (Ergo CardMedi-soft S.S, Belgium Ref. USM001 V1.0) that will be calibrated before each test session with a standard gas of known concentration and volume. Breath-by-breath gas exchange data will be measured continuously during exercise and averaged every 60 s. Peak oxygen uptake will be defined as the highest oxygen uptake (VO_2peak_) value attained toward the end of the test. Achievement of true peak effort will be assumed in the presence of two or more of the following criteria: (1) volitional fatigue (> 18 on the BORG scale); (2) peak respiratory exchange ratio (VCO_2_/VO_2_) ≥ 1.1; (3) achieving > 85% of age-predicted HR_max_; and (4) failure of VO_2_ and/or HR to increase with further increases in work rate [9]. A self-reported Borg rating of perceived exertion (scale of 6–20) will be recorded at the end of each stage. BP will be measured every 2 min throughout the test. Ventilatory thresholds (i.e. VT1 and VT2) will be assessed by standardized methods using the V-slope and ventilatory equivalents. The first ventilatory threshold (VT1) will be identified as the point of transition in the carbon dioxide production (VCO_2_) vs VO_2_ slope from < 1 to > 1, or VT1 will also be identified as the nadir of the ventilatory equivalent (VE) of VO_2_ vs work rate relationship. The second ventilatory threshold (VT2) will be identifiable as the nadir of the VE/VCO_2_ vs work rate relationship. [9] After completion of the test, participants will remain on the bike for a further 5 min for recovery with an electrocardiogram (ECG) and BP monitoring. Absolute and relative indications for terminating the exercise test will be taken into account [37]. The identification of the two VT will determine the three different exercise intensity domains or ranges for exercise design (R1, R2, R3): (R1) light to moderate exercise intensity with HR values below VT1; (R2) moderate to high or vigorous exercise intensity with HR values between VT1 and VT2; and (R3) high to severe exercise intensity with HR values up to VT2 to peak intensity. When it is not possible to identify the VT2, exercise intensity domains will be established taking into account percentages of HR reserve, i.e. moderate intensity is defined as 65–75% of HR reserve, high intensity from ≥ 85% to < 95% of HR reserve [9].

#### Biochemical profile

Morning fasting blood samples will be obtained from each participant by experienced nursing staff at the Santiago Apóstol Hospital (Miranda de Ebro, Burgos). This procedure will permit measurements of general metabolism (renal function: urea [mg/dL], creatinine [mg/dL], glomerular filtration rate [mL/ min/body surface area]; carbohydrates metabolism: basal glucose [mg/dL], HbA1c [%], fasting insulinemia [μUI/mL], homeostatic model assessment to quantify insulin resistance, HOmeostatic Model Assessment-Insulin Resistance [insulin (μU/L) × glucose (mmol/L)/22.5]; hepatic function: aspartate transaminase [U/L], alanine transaminase [U/L], alkaline phosphatase [U/L]; blood cells: hemoglobin [g/dL], hematocrit [%], leukocytes [/uL], lymphocytes [/uL], neutrophils [/uL], and platelets [/uL]), lipid metabolism (total cholesterol [mg/dL], low-density lipoprotein cholesterol [mg/dL], high-density lipoprotein cholesterol [mg/dL], triglycerides [mg/dL]; oxidative stress: ox-LDL [U/dL]), muscular and myocardial stress (troponin T [μg/L]; total creatine kinase [U/L]; pro-b-type natriuretic peptide [pg/mL]), systemic inflammation (highly sensitive C-reactive protein [mg/L], interleukin-6 [pg/mL], tumor necrosis factor-alpha [pg/mL]) and procoagulant state (D-dimer [ng/mL], fibrinogen [mg/dL]).

#### Mediterranean diet pattern

The dietary habits of participants will be assessed using a validated 137-item food frequency questionnaire (FFQ) including eight items related to seafood [38] and a 24-h recall questionnaire. A trained nurse in face-to-face interviews and group counseling will complete the FFQ. Nutrient intakes will be computed using Spanish food composition Tables [39]. The Mediet adherence is evaluated through a 14-item Mediterranean Diet Adherence Screener (MEDAS), which consists of 12 questions on food consumption frequency and two questions on food intake habits considered characteristics of the Spanish Mediterranean diet [40]. Participants will receive the main dietary recommendations included in the PREDIMED trial [21, 41]. The major characteristics of this diet are: (1) a high consumption of cereals, legumes, nuts, vegetables, and fruits; (2) a relatively high-fat consumption, mostly provided by olive oil; (3) moderate to high fish consumption; (4) poultry and dairy products consumed in moderate to small amounts; (5) low consumption of red meats and meat products; and (6) moderate alcohol intake, usually in the form of red wine [42, 43]. Participants will be encouraged, weighed, and receive advice and nutritional counseling every two weeks to help in their compliance with the dietary recommendations and requirements.

### Intervention/attention control

The AC group will receive Mediet and regular physical exercise recommendations to keep ethical procedures regarding health. In this sense, participants will be advised to participate, without supervision, in at least 30 min of moderate-intensity dynamic aerobic exercise (walking, jogging, cycling, or swimming) 5–7 days per week [13, 44]. Participants will receive information related to HR values regarding moderate exercise intensity domains for the self-monitoring of exercise intensity and will be encouraged to keep a daily record of the performed physical activity.

Exercise groups will receive Mediet recommendations and supervised exercise:

1. HV-HIIT group: high-intensity (HR values up to VT2 to peak intensity or ≥ 85% to < 95% of HR reserve) interval training and HV increasing gradually from 20 min to 40 min and alternating high and moderate intensities at different protocols; and
2. LV-HIIT group: HIIT and low-volume (20 min) alternating high and moderate intensities at different protocols.

### Exercise intervention program

The participants in the HIIT groups will exercise two non-consecutive days per week for 16 weeks under supervision by an exercise specialist and we will also request to continue with their normal activity patterns outside of the study protocol. All the exercises sessions will start and finish with BP monitoring; training intensity will be controlled by HR monitoring (Polar Electro, Kempele, Finland) and through the rate of perceived exertion using the Borg’s original scale (6–20 points). Each session will include a 10-min warm-up with joint mobility (i.e. starting at the top of the body and working the way down, from the neck, shoulders, upper back, hips, and ankles) with continuous leg movement to facilitate the venous return and coordination exercises (e.g. toe-tapping arm circles, heel walks, high knee walk, backwards high knee skip) and a 10-min cool-down period with passive stretching exercises on the floor to ensure a progressive return to the resting values of both HR and BP. The main portion of the training session will consist of aerobic exercises (i.e. one day of the week on the treadmill and the second one on the bike) developing progressively both the volume (i.e. 20 min to 40 min in HV-HIIT, whereas in LV-HIIT the duration will always be 20 min) and intensity. The rationale of mixing bike and treadmill will be to avoid the osteoarticular impact of two treadmill days taking into account the HIIT program. Intensity will be individually tailored to HR at moderate or vigorous intensities, adjusting the speed and incline of the treadmill or the power and speed on the bike to achieve the planned target HR (Table 2). The importance of targeting moderate and high intensity will be emphasized.

**Table 2:** Intervention program for high-volume high-intensity (HV-HIIT) and low-volume high-intensity (LV-HIIT) groups on the treadmill and the bike. Volume and intensity progression

	High-intensity interval		Moderate-intensity interval		High-intensity interval		Moderate-intensity interval	
	HV-HIIT treadmill				LV-HIIT treadmill			
Weeks	Volume (min)	Intensity (%HRres)	Volume (min)	Intensity (%HRres)	Volume (min)	Intensity (%HRres)	Volume (min)	Intensity (%HRres)
1–2	8	85	12	65	8	85	12	65
3–4	12	85	13	65	8	85	12	65
5–6	16	90	14	70	8	90	12	70
7–8	16	90	19	70	8	90	12	70
9–10	16	95	24	75	8	95	12	75
11–12	16	95	24	75	8	95	12	75
13–16	16	95	24	75	8	95	12	75
	HV-HIIT bike				LV-HIIT bike			
Weeks	Volume (min)	Intensity (%HRres)	Volume (min)	Intensity (%HRres)	Volume (min)	Intensity (%HRres)	Volume (min)	Intensity (%HRres)
1–2	2–2:30	85	17:30	65	2–2:30	85	17:30	65
3–4	3–3:30	85	21:30	65	3–3:30	85	16:30	65
5–6	4–4:30	90	25:30	70	4	90	16	70
7–8	5–5:30	90	29:30	70	4	90	16	70
9–10	6–6:30	95	33:30	75	4	95	16	75
11–12	7–7:30	95	32:30	75	4	95	16	75
13–16	8	95	32	75	4	95	16	75

Several strategies will be implemented to maximize adherence, including music in all sessions, individualized attention at the intervention sessions, and telephone calls following missed sessions.

#### High-intensity interval training protocol on the treadmill

The high intensity aerobic exercise groups will carry out a 5- to 10-min warm-up period at a moderate-intensity (i.e. HR values between VT1 and VT2 or 65–75% of HR reserve) on the treadmill, before walking two intervals of 4 min at high intensity (i.e. HR values up to VT2 to peak intensity or ≥ 85% to < 95% of HR reserve). The participants will exercise at the lower-intensity limit for the first two weeks of the training period before increasing the intensity towards the upper limit. Between the high-intensity intervals, 3 min of walking at moderate intensity will be conducted. The training session will end with a 4-min cool-down period at moderate intensity. This is a traditional protocol in cardiac rehabilitation programs and research with cardiac patients [15, 45]. This will give a total exercise time of 20 min. While this protocol will be kept in the LV-HIIT group, every two weeks the HV-HIIT will progress to four intervals of 4 min at high intensity and 40 min of total volume (Table 2).

#### High-intensity interval training protocol on the bike

The high-intensity aerobic exercise groups will carry out a 5- to 10-min warm-up period on the bike. After that, the participant will cycle for 30 s at high intensity (i.e. HR values up to VT2 to peak intensity or ≥ 85% to < 95% of HR reserve) followed by 60 s at moderate intensity (i.e. HR values between VT1 and VT2 or 65–75% of HR reserve) [46]. Four repetitions (1 rep = 30 s high intensity followed by 60 s moderate intensity) will be initially performed in both groups and gradually increased to 16 repetitions in the HV group, while eight repetitions will be completed (Table 2) in the LV group. The training session will end with a 4- to 7-min cool-down period at moderate intensity. The protocol design for biking has already been established in cardiac rehabilitation programs [14].

### Participant withdrawal

A participant could leave the study at any time. When withdrawing from the study, the participant should let the research team know that he/she wishes to withdraw. The following are the trial-related reasons for the participant to be discontinued from the trial by the principal investigator: (1) failure to maintain 80% compliance to exercise training sessions or even the last four weeks (minimum eight sessions), to avoid detraining; (2) more than two consecutive weeks without training, to avoid detraining; (3) participant’s condition or disease progresses; (4) participant experiences a serious adverse event (e.g. angina, dyspnea, light-headedness) [47] that requires discontinuation or withdrawal from the study according to the study protocol; or (5) pregnancy.

### Data analysis

The primary outcome variable of this study is VO_2peak_. A priori power analysis (G*Power 3 statistical software) [48] was performed to calculate the average sample size. Due to the lack of any source on the effect of VO_2peak_ in MI patients, we speculate a similar increase based on a study of coronary artery disease [49]. Therefore, we assume a mean change of 5.0 mL kg^−1^ min^−1^ and a common standard deviation (SD) of 4.0 for participants allocated to the HIIT groups and a mean change of 3.0 ± 4.0 mL kg^−1^ min^−1^ for participants allocated to the AC group. Based on 1:1:1 randomization to three treatment arms with equal group size, we expect that pre-post intervention differences in our design would be achieved with 177 people (59 each group, α = 0.05, Cohen medium effect size f = 0.23, 80% power). Assuming a maximum loss of follow-up of 10%, the plan will be to recruit a total of 194 individuals. The statistical analysis will be performed using IBM SPSS Statistics, Version 22.0 (IBM Corp., Armonk, NY, USA). In the final analysis, four comparisons will be made: HIIT groups vs AC; HV-HIIT vs LV-HIIT; HV-HIIT vs AC; and LV-HITT vs AC. Two parametric tests will be performed after all assumptions for each test are met. For comparisons between groups at baseline, one-way analysis of variance (ANOVA) or the non-parametric method of Kruskal–Wallis and Chi-square test will be used. A linear regression model with ANOVA will be used to assess training effects on the primary and secondary study outcomes. We will examine the delta (Δ) score for each group (AC, HV-HIIT, LV-HIIT), adjusting for age, sex, changes in body mass, and the initial value of each of the dependent variables. Helmert contrasts will be performed to analyze the difference between the two exercise groups pooled together and the AC group. Bonferroni correction was used to determine the level of significance when a significant main effect was found. The differences between dropouts and participants who remain in the study will be examined; the data will be analyzed according to the intention-to-treat principle [50]. For each outcome variable, the effect size and the level of significance corresponding to the main group (between-subjects), time (within-subjects), and interaction (group × time) will be reported. To prevent type I error, post hoc comparisons (pre vs post by group) will be performed when a significant interaction effect is present. Values will be expressed as mean ± SD. The significance level will be set at 5% (α = 0.05). Practical significance will be assessed by calculating Cohen’s *d* effect size. Effect sizes (*d*) > 0.8, in the range of 0.5–0.8, in the range of 0.2–0.5, and < 0.2 will be considered as large, moderate, small, and trivial, respectively.

## Discussion

A large-scale national Swedish registry study showed that the risk of cardiovascular events remained high in the first year post MI, indicating that those patients should be managed with effective prevention programs [51]. Therefore, one of the main challenges in secondary prevention after MI is the reduction in mortality and morbidity through evidence-based interventions [7]. In patients with stable angina pectoris secondary to coronary atherosclerosis, the review found that interval training improved cardiorespiratory fitness, endothelial and ventricular function, and morphology to a greater degree than conventional light- to moderate- and moderate- to high-intensity continuous aerobic training [9]. On the other hand, less is known regarding the effects of LV-HIIT, but growing evidence suggests this type of training stimulates physiological remodeling comparable with moderate-intensity continuous training despite a substantially lower exercise volume [17]. A recent study has shown that LV-HIIT exercise elicits similar cytokine and oxidative stress responses to steady-state at a high intensity higher volume exercise [52]. However, based on the review of the literature, several issues are still open to question and would benefit from further investigation in MI population:

- Does LV-HIIT provide similar benefits compared to HV-HIIT and is it associated with a lower risk of all-cause mortality in population after MI, being able to confirm that “less is more”?
- Which combination of treatment (Mediet recommendations + physical activity recommendation vs Mediet recommendations + supervised exercise) is most valuable in reducing cardiovascular risk in patients after MI?

The increase in the prevalence of heart disease and related co-morbidities is often associated with polypharmacy and disabilities, such as physical and emotional frailty. Non-pharmacologic management strategies including regular exercise and a healthy diet are necessary to get a comprehensive care plan for the whole disease. This study will be the first clinical trial comparing the effects of two different volumes of HIIT programs with Mediet recommendations for people after MI and also compared to an attention control group receiving only recommendations. The results of this trial will provide good evidence for physical rehabilitation in this population and what type of treatment achieves better physical results.

### Trial status

Recruitment for the trial is finishing at the time of submission.

## Additional file

Additional file 1: SPIRIT 2013 Checklist: Recommended items to address in a clinical trial protocol and related documents. (DOC 121 kb)

## Abbreviations

AC: Attention control
AMI: Acute myocardial infarction
BP: Blood pressure
CIMT: Carotid intimae media thickness
CPET: Cardiopulmonary exercise test
CVD: Cardiovascular diseases
DBP: Diastolic blood pressure
ECG: Electrocardiogram
FFQ: Food Frequency Questionnaire
FITT principle: Frequency, intensity, time or volume, and type or modality
FMD: Flow-mediated dilation
HADS: Hospital Anxiety and Depression Scale
HDL: High-density lipoprotein cholesterol
HIIT: High-intensity interval training
HR: Heart rate
HV-HIIT: High-volume and high-intensity interval training
IPAQ: International Physical Activity Questionnaire
LDL: Low-density lipoprotein cholesterol
LV-HIIT: Low-volume and high-intensity interval training
MEDAS: Mediterranean Diet Adherence Screener
Mediet: Mediterranean diet
MI: Myocardial infarction
MSWT: Modified Shuttle Walking Test
SBP: Systolic blood pressure
SF-36: Short Form Medical Outcome Questionnaire
VCO_2_: Carbon dioxide production
VO_2_: Oxygen uptake
VO_2peak_: Peak oxygen uptake
VT: Ventilatory threshold
VT1: First ventilatory threshold
VT2: Second ventilatory threshold
